# Supplementary material for: MicroRNA as Possible Mediators of the Synergistic Effect of Celecoxib and Glucosamine Sulfate in Human Osteoarthritic Chondrocyte Exposed to IL-1β
Source: Int J Mol Sci. 2023 Oct 8;24(19):14994. doi: 10.3390/ijms241914994 (PMC10573984; doi:10.3390/ijms241914994)
Supplement: Supplementary file 1 [file ijms-24-14994-s001.zip › ijms-2625067-supplementary.pdf]

MicroRNA as Possible Mediators of the Synergistic Effect of Celecoxib and Glucosamine Sulfate in Human Osteoarthritic Chondrocyte Exposed to IL-1β

Supplementary material

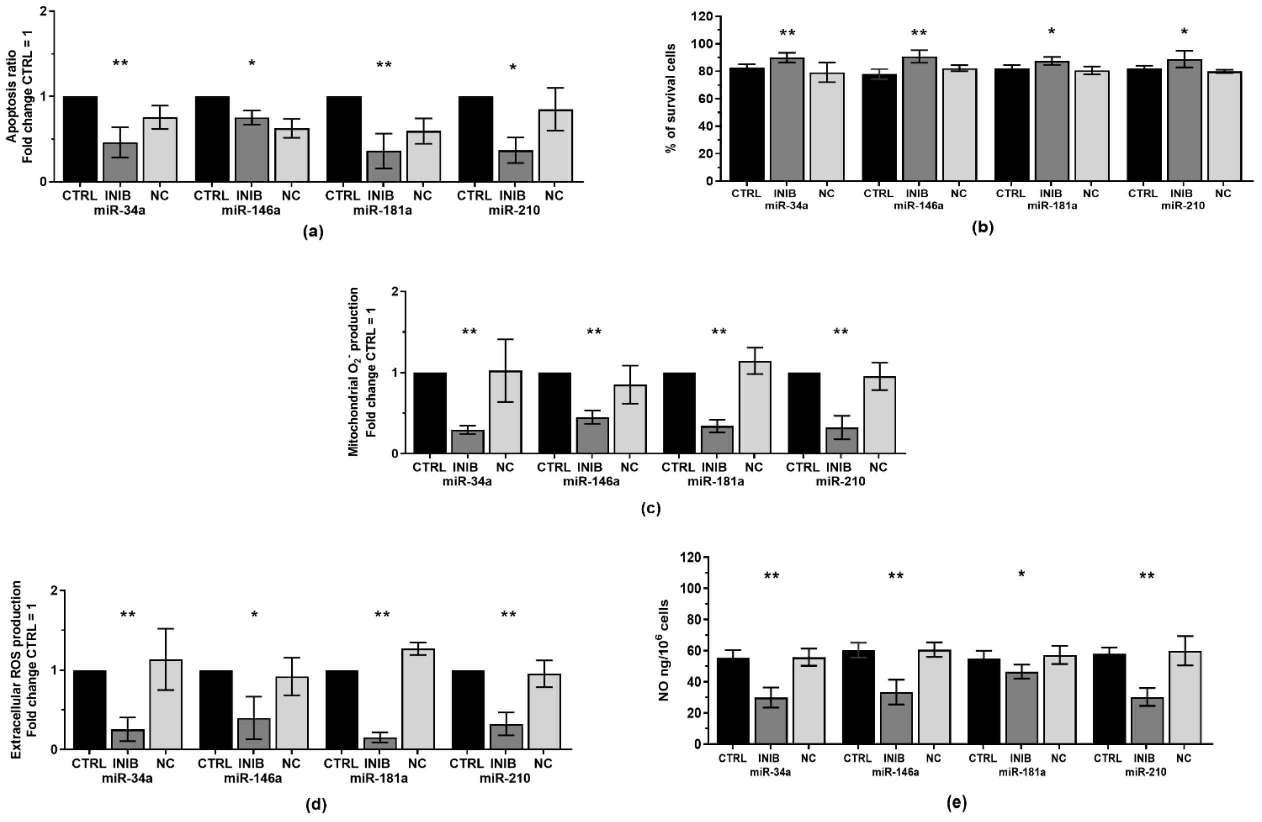

Figure S1. Efficacy of miRNA silencing on apoptosis, viability, and redox balance. Chondrocytes were evaluated at control (CTRL) condition, after 24 h of transient transfection with miR-34a, miR-146a, miR-181a, and miR-210 inhibitors (50 nM) or NC (5 nM). (**a**,**c**,**d**) Apoptosis detection and reactive oxygen species (ROS) production by flow cytometry. The results were expressed as fold change of the value of interest respect to CTRL, reported equal to 1. (**b**) Viability by MTT assay (**e**) Nitric oxide (NO) release by Griess method. Data were represented as mean ± standard deviation. **p* < 0.05, ***p* < 0.01 versus CTRL or NC.

Table S1. List of primers for quantitative real time PCR.

Target Genes	Cat. No. (Qiagen)
BCL2	QT00000721
NRF2	QT00027384
miR-34a	MS00003318
miR-140	MS00003319
miR-146a	MS00003535
miR-155	MS00008778
miR-181a	MS00006692
miR-210	MS00006782
miR-375	MS00003741
miR-let7e	MS00031801
ACTB	QT00095431
SNORD-25	MS00014007

Abbreviations: BCL2 = B-cell lymphoma 2, NRF2 = nuclear factor erythroid 2 like 2, miRNA = microRNAs, ACTB = Actin Beta, SNORD-25 = Small Nucleolar RNA, C/D Box 25.
